# Structural and Functional Organization of the Root System: A Comparative Study on Five Plant Species

**DOI:** 10.3390/plants9101338

**Published:** 2020-10-10

**Authors:** Adriano Sofo, Hazem S. Elshafie, Ippolito Camele

**Affiliations:** 1 Department of European and Mediterranean Cultures: Architecture, Environment and Cultural Heritage (DiCEM), University of Basilicata, Via Lanera, 20, 75100 Matera, Italy; 2 School of Agricultural, Forestry, Food and Environmental Sciences (SAFE), University of Basilicata, Via dell’Ateneo Lucano 10, 85100 Potenza, Italy; hazem.elshafie@unibas.it (H.S.E.); ippolito.camele@unibas.it (I.C.)

**Keywords:** litter decomposition, root development and morphology, root-soil continuum, soil C/N, soil microorganisms, tea bag index

## Abstract

Plants are affected by soil environments to the same extent that they affect soil functioning through interactions between environmental and genetic factors. Here, five plant species (broad bean, pea, cabbage, fennel, and olive) grown under controlled pot conditions were tested for their ability to differently stimulate the degradation of standard litter. Litter, soil C and N contents were measured for evaluating chemical changes due to plant presence, while soil microbial abundance was evaluated to assess if it had a positive or negative catalyzing influence on litter decomposition. The architecture and morphological traits of roots systems were also evaluated by using specific open-source software (SmartRoot). Soil chemical and microbiological characteristics were significantly influenced by the plant species. Variations in soil C/N dynamics were correlated with the diversity of root traits among species. Early stage decomposition of the standard litter changed on the basis of the plant species. The results indicated that key soil processes are governed by interactions between plant roots, soil C and N, and the microbial metabolism that stimulate decomposition reactions. This, in turn, can have marked effects on soil chemical and microbiological fertility, both fundamental for sustaining crops, and can promote the development of new approaches for optimizing soil C and N cycling, managing nutrient transport, and sustaining and improving net primary production.

## 1. Introduction

Plants are affected to the same extent that they affect soil systems through interactions between environmental and genetic factors [1,2,3]. Plant traits have profound effects on soil fertility, net primary productivity, soil decomposition processes, soil nutrient cycling, and soil organic matter (SOM) levels [3,4,5]. Litter abundance and type have a significant role in seedling recruitment and can provoke the abundance of some plant species instead of others, determining plant species composition [6,7,8].

The diverse chemical composition of litter is a potentially important functional trait affecting its decomposition. Additionally, litter composition has relevant effects on soil organisms, with different litter types promoting different subsets of soil microflora and fauna, and consequently, different litter decomposition rates [9,10,11,12]. Furthermore, litter N and C contents are the other two key factors in regulating litter decomposition, with both often acting as limiting factors [13,14,15]. On the other hand, root traits can influence the decomposition process [16]. Among the plant traits, the root system architecture (growth, development, and density) and chemical composition of root exudates cause changes in the soil physicochemical properties, the composition of soil microbial communities, and litter decomposition [17]. This shows the importance of vegetation-soil feedbacks and soil–roots interactions that are regulating litter decomposition [4]. These feedbacks and interactions make the root–soil system as a continuum where influences are mutual and bidirectional.

Considering that each plant species has a specific root architecture and function, the importance of plant community composition in controlling the soil decomposition processes is today well-known through its influence on litter quality, which affects mass loss and nutrient release [2,3]. Research has focused on the effects of different species on litter quality and decomposition, but in many cases, it used non-standardized litter [2,3,17,18] or different kinds/mixtures of it [14,15]. In other cases, experiments were conducted in native environments [10,15,19] where soil types and climatic conditions can strongly affect litter decomposition, and abiotic factors are not kept under control. This is a key point, as the climate is another important factor influencing litter decomposition, especially in terms of soil moisture and temperature [14]. In all these studies, plant species of agronomic importance have never been considered.

A reliable, new and relatively simple way for conducting experiments on decomposition occurring in the soil is to evaluate litter decomposition by the tea bag index (TBI) method, which measures the decay of plant material by using two types of tea bags (green and red tea) as standard plant material [20,21]. This method has been successfully used for evaluating early stage litter decomposition in different environments, and it was found that this parameter is affected by various factors such as soil type, land use, soil temperature, and soil moisture [22,23].

Here, five plant species were tested for their ability to differently stimulate the degradation of standard litter. The initial and final litter carbon (C) and nitrogen (N) contents were measured, and the architecture and morphological traits of the roots systems were also evaluated by using specific open-source software. Comparisons between the root system architecture, plant epigean, and hypogean biomass, litter decomposition, and soil microbial abundance were determined in five cultivated species to understand: (a) if plants can differently modulate early stage litter decomposition (labile SOM); (b) the role of biomass and root traits in the litter decomposition process; and (c) the contribution of soil microorganisms in determining the litter decomposition rates. We hypothesized that the choice of the species to cultivate such as in crops forming monocultures and/or during cultural rotation significantly affects litter decomposition. The dynamics of litter decomposition regulate the pathways to the formation and stabilization of SOM. This, in turn, can have marked effects on soil chemical and microbiological fertility, both fundamental for sustaining crops and maintaining soil health and quality.

## 2. Results

### 2.1. Plant Traits and Root Image Processing

The fresh weight of the shoots significantly differed among the species, and these changes were paralleled by the trends in root fresh weight (Table 1). Olive, because of its larger size compared to the other plant species, showed the highest values of both shoot and root dry weight (DW) (1.242 and 937 g, respectively) and root/shoot DW (0.68) (Table 1). The values of root/shoot DW ratio in olive plants were in accordance with those found by Sofo et al. [24] in well-irrigated two-year-old plants (Table S1). Olive also had the highest values of the shoot and main root length, with a main root/shoot length of 0.26, similar to that of broad bean (0.23 cm) (Table S1). The main root/shoot length was comparable in pea and fennel, while the highest value was found in cabbage (0.96 cm) (Table S1).

All the parameters measured and calculated by SmartRoot software are reported in Table 1. Except for specific root length, the values were significantly higher in olive and, among the herbaceous species, in broad bean. Marked differences among species were found for total root length, the total length of lateral roots, number of root tips, and total root surface area. The values of the main root length measured by SmartRoot (Table 1) were 8–25% higher than those measured manually (Table S1), but followed the same trend. A high development of lateral roots, in terms of total length and root tips, was found in broad bean (0.45 m · g^–1^ fresh weight (FW) and 1151, respectively), followed by pea (0.45 m · g^–1^ FW and 713, respectively). These variations in lateral root development among species are due to both genetic traits and phytohormonal balance, particularly the auxins/cytokinins ratio [25]. Broad bean and pea showed equally dense (0.45 m · g^–1^ FW), well-developed lateral roots compared to the other herbaceous species (Table 1), and a nodulated root structure (Figure S1a,b). Except for the fennel, whose total root length and, consequently, total root surface area were low (5.34 m and 0.04 m^2^, respectively), average root diameter and total root surface area in the other species followed a similar trend.

### 2.2. Standard Litter Decomposition

The weight differences of the tea inside the two types of the tea bags (green and red) allowed us to calculate the decomposition indices (Table 2). Green tea decomposed faster than red tea, indeed the fraction of remaining green tea (*X*_g_) was lower than the fraction of remaining red tea (*X*_r_) in all tea samples (Table 2). Among these, the highest value of the fraction of remaining green tea (*X*_g_) was found in olive (0.719), followed by broad bean, fennel, pea, and cabbage, whereas the fraction of remaining red (*X*_r_) was significantly higher in olive (0.719), followed by broad bean, pea, fennel, and cabbage (Table 2). On the other hand, red tea decomposed much slower (*X*_r_), so after three months, it was still in the first phase of decomposition. With this method, in only three months, we had a long temporal scale to evaluate litter decomposition dynamics.

From the tea bag weights (Table 2), it was possible to determine how much of the labile fraction of the material was decomposed and how much was stabilized (stabilization factor, *S*). Low values of *S* indicated a higher and further decomposition of the labile fraction, compared to higher values [20]. This parameter resulted in being significantly higher in olive (0.666), followed by broad bean, fennel, and pea (not significantly different from each other), and then cabbage (Table 2). High fractions of *X*_r_ cause lower initial decomposition rates (*k*), resulting in a slower decomposition [20,26]. The decomposition rate constant (*k*) showed a reverse trend compared to *S*, with the lowest value (0.010) in olive, followed by broad pea, cabbage, pea, and the highest (0.029) in fennel.

### 2.3. Soil Chemical Analysis

The values of litter organic carbon (LOC) were not significantly different among plant species, except for green tea in cabbage (minimum value of 24.03 g · kg^−1^), while they were all significantly lower compared to the control (Table 3). The levels of litter total nitrogen (LTN) remained significantly high for broad bean and pea, causing low values of LOC/LTN (Table 3). Generally, the trends of LOC, LTN, and LOC/LTN in green and red tea litter were similar (Table 3).

The presence of plants caused a decrease in soil organic carbon (SOC) that was less marked in olive (35.41 g kg^−1^ vs. 38.54 g kg^−1^ in initial soil) (Table 3). This result was probably linked to the low and slow litter decomposition in olive soil (Table 2). Soil total nitrogen (STN) remained high in broad bean (3.55 g · kg^−1^) and pea (3.62 g · kg^−1^) and unchanged in the control, compared to the soil before planting (Table 3).

**Figure 1:**
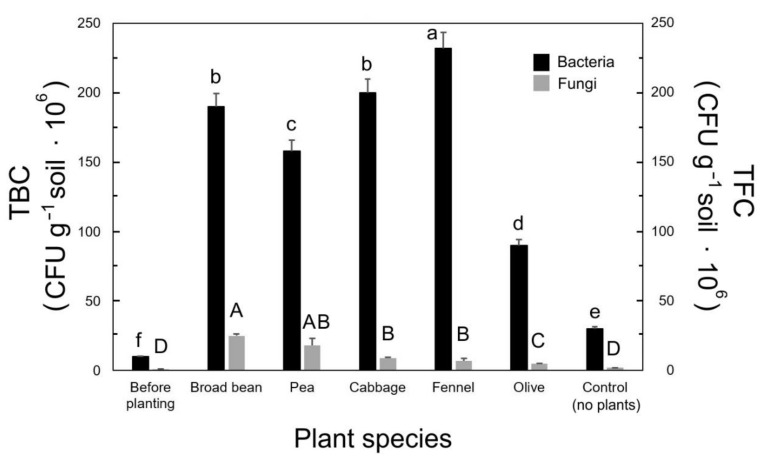
Total bacterial count (TBC) and total fungal count (TFC) from soils with different plant species. Each value represents the mean (±SD) of five replicates (composite soil samples; *n* = 5) for each species. The values followed by different letters (a–f; lowercase for TBC and uppercase for TFC) are statistically different (*p* ≤ 0.01).

Soil pH was influenced by plant presence and species, as shown in Figure 1. The lowest pH was found in fennel, whereas olive did not statistically differ by the control and the soil before planting (Table 3). The values of soil pH for broad bean, pea, and cabbage were not statistically different (Table 3).

### 2.4. Microbial Counts

The obtained results from the TBC assay showed that there were significant differences in bacterial abundance between the control (30 × 10^6^ CFU g^−1^) and all other plant–soil samples (Figure 1). The highest number of bacterial colonies was observed for fennel (200 × 10^6^) followed by cabbage, broad bean, and pea, which were 200 × 10^6^, 190 × 10^6^, and 158 × 10^6^ CFU g^−1^, respectively (Figure 1). In the case of olive, 90 × 10^6^ CFU g^−1^ was the lowest value (Figure 1). The soil before planting had very low bacterial colonies (10 × 10^6^ CFU g^−1^), compared to the other soil samples. Regarding fungi (TFC), a high number of fungal colonies was observed in broad bean (25 × 10^6^ CFU g^−1^), followed by pea (17 × 10^6^ CFU g^−1^), whereas cabbage, fennel, and olive showed low fungal abundance (9 × 10^6^, 7 × 10^6^, and 5 × 10^6^ CFU g^−1^, respectively), with the control without plants at 2 × 10^6^ CFU g^−1^ (Figure 1). The soil before planting had very low fungal colonies (1 × 10^6^ CFU g^−1^) compared to the other plant–soil samples (Figure 1).

## 3. Discussion

All higher plants, defined as rhizophytes, are characterized by the structure variability of roots, though to a different degree [4,27,28]. In this study, particularly interesting was the case of fennel, with a root/shoot DW ratio of 0.17 due to the limited taproot and the swollen bulb-like stem base, which is not considered part of the root (Figure S2). A very different root morphology was found in broad bean and pea, compared to cabbage (Table S1 and Figure S2). Indeed, cabbage is characterized by having a thin, deep, and dense taproot (Figure S2), as assessed by the values of root/shoot DW (0.36) and main root/shoot length (0.96) (Table S1). On the other hand, *Leguminosae* has relatively shallow root systems [29] (Figure S2). Indeed, the main root/shoot length in broad bean and peas was 0.23 and 0.19, significantly lower than that of cabbage (Table S1). Some root traits (root DW, root total length, and total root surface area) were significantly and positively correlated to the main litter decomposition (*S* and *k*) (Table S2), thus demonstrating that species-specific root architecture can influence the dynamics of SOM turnover. Links between plant species diversity, litter chemistry, and soil functions such as soil respiration, net N mineralization, and microbial biomass have been found by Meier and Bowman [16]. They highlighted that litter chemical composition, mainly depending on plant species diversity, can be a potentially important functional trait affecting decomposition dynamics in a specific soil. Cornelissen [18] reached similar conclusions in an experimental multispecies screening of leaf decomposition using various plant species groups, finding a correlation between plant taxonomy and litter decomposition rates. On a broader scale, Cornwell et al. [4] pointed out strong connections between whole-plant carbon strategy, SOM decomposability, and biogeochemical cycling (particularly C and N), that are crucial for understanding both vegetation–soil feedbacks, and for improving forecasts of the global carbon cycle.

An innovative and standardized way to measure litter decomposition is the tea bag index (TBI) method, which measures the decay of plant material by using two types of tea bags (green and red tea) [20,21]. Based on TBI, tea is used as a standard litter [21]. Compared with more sophisticated and expensive methods such as measuring fluxes of carbon in the decomposition of isotopically labeled substrates, the TBI method is cheap and handy [30]. The values of the two main indices of the TBI (*S* and *k*) obtained in this study (Table 2) indicated that the decomposition of the labile fraction of the litter was high (*S*) and moderately fast (*k*) in soils covered by cabbage, moderately high (*S*), and moderately fast (*k*) in broad bean and pea, moderately high (*S*) and fast (*k*) in fennel, and low (*S*) and slow (*k*) in olive (Table 2). Finally, tea bags in the control with no plants decomposed little (*S*) and very slowly (*k*) (Table 2). From the morphometric data of Table S1 and the correlation analysis reported in Table S2, it appears that root depth/length governed litter decomposition in cabbage, lateral roots in broad bean and pea, and the structure of the hypogean part (swollen bulb + roots) in fennel, whereas the slow litter decomposition in olive soil was linked to low values of specific root length and high root diameter.

The observed high values of STN in broad bean and peas were likely due to the N-fixing capacity of *Rhizobium* living in *Leguminosae* roots [13,15,31], which enriches the soil with N. The soil N content is particularly important, as a higher soil N availability increases the N levels in the litter, which in turn increases the decomposition rates and litter N release, creating a positive feedback loop to litter N [5]. Except for olive, the values of SOC/STN were significantly lower than those found in the soil before planting (Table 3). According to Hobbie [5] and Meier and Bowman [16], soil C and N cycling during decomposition are controlled by the composition and diversity of the chemical compounds released by roots into the soil, which were different among plant species. Many of these compounds are acids and can influence litter decomposition, as also found in the correlation analysis, where soil pH was significantly related to *k* (Table S2). Moreover, all the plant species, except olive, reduced soil pH (particularly fennel) due to root acid exudates (Table 3), thus contributing to litter decomposition. Osanai et al. [15] demonstrated that plant species identity had a substantial impact on both litter decomposition and N cycling via both litter chemistry and specificity of the associated soil microbial community. On this basis, changes in botanical composition can alter decomposition and nutrient release, altering ecosystem productivity and carbon sequestration potential [15].

Doubtless, there is a strict relationship between soil microbial abundance and litter decomposition [9,11,16], as also shown by the correlation analysis, where both *S* and *k* were significantly affected by TBC and TFC (Table 2). In olive soil, in which litter decomposition was low and slow (Table 3), both bacteria and fungi were found to be significantly lower than in the other soils, except for the controls before planting and with no plants (Figure 1). Fennel, with the faster litter decomposition (Table 3), presented the highest bacterial count (Figure 1). A certain enrichment in soil bacteria was observed in the soils without plants compared to the initial control (Figure 1). This notwithstanding, it appears clear that the presence of roots and the relative rhizospheric microenvironments caused a higher microbial abundance, likely due to root inorganic and organic exudates, physical changes in soil structure and aggregation, pH variation, root cell decomposition, and consequent increases in soil organic matter and nutrients, and many other physicochemical and biological factors [2,3,5,10]. Ayres et al. [11] demonstrated conclusively that the abundance and composition of soil microbial communities specialized in decomposing the litter strongly depends on the plant species present in that soil. Moreover, Bray et al. [3] found that the initial plant community and litter chemistry determine the rate of decomposition and microbial community composition in the early stages of decomposition.

In conclusion, soil chemical and microbiological characteristics were significantly influenced by the plant species. Variations in soil C/N dynamics were correlated with the diversity of root traits among species. Litter decomposition potential evaluated using standard tea bags changed with the plant species cultivated. Generally, a deep-rooted plant (main root length) with a dense root system (evaluated by the value of specific root length), like cabbage, was able to strongly increase soil decomposition (*S*), but great influence on litter decomposition has also been observed in N-fixing *Leguminosae* such as broad bean and pea (with well-developed lateral roots), which is able to significantly influence soil N and C/N ratios. On the other hand, the speed of litter decomposition (*k*) was mainly regulated by high hypogean biomass, as in the case of fennel (with a conspicuous underground shoot), and less influenced by the other root parameters. Finally, a tree species like olive was much less able to affect litter decomposition rate and speed, also considering its high total root length and total root surface area. Aside from root architecture, modifications in soil pH and different microbial abundances were the key parameters able to influence litter decomposition. These results support the idea that key soil processes are regulated by interactions between plant roots, soil C and N, and the microbial metabolism that catalyzes decomposition reactions. Knowledge about plant species and the relative litter dynamics may be useful for modeling soil decomposition rates under natural vegetation or cultivated fields differing in species/crop composition. Finally, we are confident that this study produces applicable knowledge, and can induce farmers to leave crop residues on the field after harvest (e.g., roots, that are often removed at the end of the season) and let them decompose. This practice could increase soil organic matter stock and ameliorate soil chemical and microbiological fertility, promote the development of new approaches for optimizing soil C and N cycling, manage nutrient transport, and sustain and improve net primary production.

## 4. Materials and Methods

### 4.1. Experimental Site, Orchard Management, and Soil Sampling

The experimental area (Trani, BT, Puglia Region, Italy; 41°16′25″32N, 16°24′58″32E) is characterized by a semi-arid climate, with an annual rainfall of 595 mm (mean 1995–2019) and a mean annual temperature of 16.0 °C. The trial was carried out outdoor in the autumn–winter 2019–2020 (November–February). The experiment was performed using the same soil type and under the identical climatic conditions. This allowed eliminating any indirect effects due to initial leaf litter composition, soil type, or climate regime. On 1 November, 2019, 2-week-old seedlings of broad bean (*Vicia faba* L.), pea (*Pisum sativum* L.), cabbage (*Brassica oleracea* L., cv. capitata), fennel (*Foeniculum vulgare* L.), and one own-rooted two-year-old plant of olive (*Olea europaea* L., cv. Coratina) were grown uniformly outdoors in each of the 30-L rectangular (conic for olive) pots filled with the same mixture of loam, peat, and sand (in the proportion of 1:1:1). The studied speces were chosen on the basis of their different habitus and root architecture and morphology. Five pots for each plant species were used, with a total of 10 plants for each plant species (two plants per pot). Only for olive was one plant per pot placed, with a total of five plants. Five control pots with only soil and with no plants were kept as a control. Soil water content was maintained at a constant value of around 85% of the water holding capacity of the pot by integrating the amount of water lost through evaporation and transpiration during the day.

### 4.2. Plant Traits and Root Architecture

On 26 February, 2020, at the end of the experimental trial, five plants per species (one for each pot, randomly chosen) were carefully extracted from the soil and photographed. Shoot and root fresh weight and their main length were measured with a digital scale and a ruler, respectively (Figure S2). Then, roots were gently washed under tap water to not damage the fine roots, and the entire root system was immediately scanned at high resolution (6400 DPI) with Epson Perfection V850 Pro (Epson Ltd.; Suwa, Japan). Both shoots and roots were dried at 98 °C until a constant weight was achieved (48 h) for the determination of dry weight.

For each plant species, random portions of roots (Figure S1) were analyzed by SmartRoot (https://smartroot.github.io/) [32], a semi-automated root image analysis freeware software. For each species, the following root morphological measurements were made: total root length, main root length, total length of lateral roots, specific root length, number of root tips, average root diameter, and total root surface area. The values of these parameters were averaged from five root portions per plant and then normalized on a fresh weight-basis for the whole root.

### 4.3. Standard Litter Decomposition

#### 4.3.1. Tea Bags Installation and Final Sampling

On 28 November 2019, one green tea bag (*Camelia sinensis*; n. EAN 87 10908 90359 5; Lipton) and one rooibos tea (red tea) bag (*Aspalanthus linearis*; n. EAN 87 22700 18843 8; Lipton Unilever, Glasgow, UK) were air-dried, weighed (including bag, cord, and label) and inserted 15 cm apart from each other at a 10 cm soil depth using a planting spade at each pot (*n* = 5) for each plant species. The chosen spots were near those chosen for the microbiological analyses. The tea bags used were of the non-woven type. The string and the label were left above the ground to facilitate subsequent retrieval. The pits were closed using the same removed soil, and the positions marked.

The tea bags were retrieved after 90 days, on 26 February 2020 (Figure S3). Soil parts and roots were removed and the tea bags oven-dried at 50 °C for 48 h and then placed in a desiccator until reaching a constant weight. After drying, the soil attached to the surface of tea bags was carefully removed with hands and a slight brush, and the final weight (including bag and cord, but not the label) was recorded.

#### 4.3.2. Calculation of Decomposition Index

From the weight differences calculated from both the green and red tea bags (Table 2), two main indices were calculated according to the model of Keuskamp et al. [20], namely the decomposition rate constant (*k*) and the stabilization factor (*S*) (i.e., the inhibiting effect of environmental conditions on the decomposition of the labile fraction). While *k* can only be estimated from the early stages of decomposition (i.e., from red tea data after three months), *S* is related to the limit value and is estimable after most of the labile material (i.e., green tea) decomposed. Both *k* and *S* were calculated following these equations (Equation (1)):

*X*_t_ = *a*e^−*kt*^ + (1 − *a*) (1)

where *X*_t_ is the weight after the incubation time *t* in days (90 days); *a* is the labile fraction (green tea); and (1 – *a*) is the recalcitrant fraction (red tea) of the litter (Equation (2)):

*S* = 1 − (*a*_g_ / *H*_g_) (2)

where *a*_g_ is the decomposable fraction of green tea and *H*_g_ is the hydrolyzable fraction of green tea.

The decomposable fraction of red tea (*a*_r_) was calculated from the hydrolyzable fraction of red tea (*H*_r_) and the stabilization factor *S* (Equation (3)):

*a*_r_ = *H*_r_ (1 − *S*) (3)

with *X*_t_ of the red tea (*X*_r_) and *a*_r_ known, *k* can be calculated using Equation (1).

### 4.4. Litter and Soil Chemical Analysis

The dry tea samples described above were removed from the bag, ground, and used for C and N chemical analyses. On 26 February 2020, soil sampling was performed in all of the pots. For each plant species and the control, five composite samples (*n* = 5) were prepared. Sub-samples were picked at three points of the pot (two lateral and one central; soil depth = 10 cm) and pooled on-site to make up a composite soil sample of about 250 g. These sampling techniques allowed for soil heterogeneity to be minimized, according to Sofo et al. [31]. After removing the visible plant residues, the soil composite samples were immediately stored in sterilized plastic bags at 4 °C for chemical measurements and subsequently analyzed within three days. Soil samples were air-dried at approximately 25 °C and then sieved through a 2-mm stainless steel sieve. The size fraction smaller than 2 mm was used for soil chemical analyses. Litter and soil C/N analysis were also performed before incubation/planting (28 November 2019) and in the control with no plants. For each plant species, five replicates of tea and composite soil samples (*n* = 5) were prepared.

All the soil samples were dried at 105 °C for 24 h, placed in a desiccator until a constant weight was reached, and then sieved through a 2-mm stainless steel sieve. The size fraction smaller than 2 mm was used for soil chemical analyses. Soil pH was measured by a glass electrode (Basic 20; Crison Instruments SA, Barcelona, Spain) in distilled water, using a suspension 1:2.5 soil to liquid phase ratio [33]. The levels of C and N in tea and soil were determined both before and after incubation into the soil/planting. Litter organic carbon (LOC), soil organic carbon (SOC), litter total nitrogen (LTN), and soil total nitrogen (STN) were measured. The LOC and SOC level were determined by the Walkley and Black method by oxidation at 170 °C with potassium dichromate (K_2_Cr_2_O_7_) in the presence of sulfuric acid (H_2_SO_4_), and the excess K_2_Cr_2_O_7_ was measured by Möhr salt titration [33], while LTN and STN were measured by the Kjeldahl method [33].

### 4.5. Total Bacterial and Fungal Enumeration

In the same soil samples used for the C and N chemical analyses, total bacterial count (TBC) and total fungal count (TFC) were carried out using the Plate Count Agar (PCA) method. PCA, also called Standard Methods Agar (SMA), is a microbiological growth medium commonly used to assess or to monitor “total” or viable microbial cell growth of a sample. PCA was prepared in sterile distilled water (*w*/*v*): tryptone 5 g · L^–1^, yeast extract 2.5 g · L^–1^, glucose 1 g · L^–1^, and agar 15 g · L^–1^. The pH was adjusted to neutral 0.7 at 25 °C. For TFC, Potato Dextrose Agar (PDA) media was used by adding 39 g of PDA in 1 L of distilled water.

The two media were autoclaved after that at 121 °C for 20 min. Then, 10 mg L^–1^ of ampicillin and 15 mg L^–1^ of streptomycin antibiotics, sterilized by microfiltration (0.22 µM), were added at 45 °C in the case of PDA media. The plates were prepared by pouring 14 mL in each 90 mm Petri dish. The soil samples were suspended in sterile distilled water (1 g soil in 9 mL water), shaken for 15 min, and maintained at 4 °C for 60 min [34]. The soil suspensions were applied for subsequent decimal dilutions ranged from 10^–2^ until 10^–5^. 

An aliquot of 100 μL from each dilution was inoculated on each prepared media [35] following the scheme of preparation of ISO 2293 [36]. The plates were cultured manually by spreading the applied suspension amount over the agar surface. Then, all plates were incubated for 2–448 h at 30 °C TBC or 22 ± 2 °C in the case of TFC. After incubation, Petri plates containing between 30 and 300 colonies were selected for total CFU (colony-forming unit) counting. Plates with over 300 colonies could not be counted and were designated as too many to count, and those had fewer than 30 colonies were designated as too few to count. The calculation of the total number of bacteria or fungi (CFU) per g of soil was performed by dividing the number of colonies by the selected dilution factor multiplied by the amount of specimen added to liquefied agar (100 µL). As for C and N determination, both TBC and TFC were determined both before planting and in the control with no plants.

### 4.6. Statistical Analysis

The statistical analysis of the root morphology, litter/tea decomposition, and microbial abundance was performed using Sigmastat 3.1 SPSS Inc. software (SPSS Inc., Quarry Bay, Hong Kong, China). The means of all the measured parameters were treated by one-way analysis of variance (ANOVA) with the plant species type as a factor. Means were separated according to Fisher’s least significant difference (LSD) test at *p* ≤ 0.01 [37]. Five analytical replicates for each treatment (*n* = 5) were considered for each parameter measured. Correlation analysis was performed to determine the relationship between the measured parameters, computing the Pearson correlation coefficients (*R*) as a parametric measure of the linear relationship between the variables, selecting the best fits that minimized the absolute sum of squares (*R*^2^ and significance measured at *p* ≤ 0.05 and *p* ≤ 0.01) (Table S2).

## Figures and Tables

**Table 1:** Root morphological parameters measured by SmartRoot software in root portions of different plant species. Each value (±SD) represents the mean of five replicates (plants; *n* = 5) for each species. Five root portions were considered for each plant. The values followed by different letters (a–e) are statistically different (*p* ≤ 0.01) within columns.

Plant Species	Total Root Length (m)	Main Root Length (m)	Total Length of Lateral Roots (m)	Specific Root Length (m · g^−1^ FW)	Root Tips (Number)	Average Root Diameter (mm)	Total Root Surface Area (m^2^)
Broad bean	24.59 ± 3.09 b	0.17 ± 0.09 b	24.41 ± 3.21 b	0.45 ± 0.10 ab	1151 ± 88 b	3.24 ± 0.32 a	0.25 ± 0.02 b
Pea	15.24 ± 1.53 c	0.20 ± 0.04 b	15.04 ± 3.85 bc	0.45 ± 0.03 b	713 ± 34 c	2.53 ± 0.15 b	0.12 ± 0.03 c
Cabbage	11.31 ± 3.00 cd	0.55 ± 0.13 a	10.76 ± 2.09 c	0.58 ± 0.07 a	529 ± 39 d	2.07 ± 0.17 c	0.07 ± 0.02 d
Fennel	5.34 ± 0.79 d	0.15 ± 0.05 b	5.19 ± 1.77 d	0.33 ± 0.04 c	403 ± 12 e	2.48 ± 0.20 b	0.04 ± 0.01 e
Olive	127.83 ± 20.11 a	0.67 ± 0.16 a	127.16 ± 26.79 a	0.28 ± 0.08 c	11965 ± 937 a	5.05 ± 0.17 a	6.78 ± 0.34 a

**Table 2:** Mass loss of green and red tea, fraction of remaining green (*X*_g_) and red tea (*X*_r_), stabilization factor (*S*), and decomposition rate constant (*k*) calculated from the green tea and red tea bags. Each value represents the mean (±SD) of five replicates (green tea/red tea bags five; *n* = 5) for each species. The values followed by different letters (a–d) are statistically different (*p* ≤ 0.01) within columns.

Plant Species	Green Tea Mass Loss (g)	Fraction of Remaining Green Tea (*X*_g_)	Red Tea Mass Loss (g)	Fraction of Remaining Red Tea (*X*_r_)	Stabilization Factor (*S)*	Decomposition Rate Constant (*k)*
Broad bean	0.521 ± 0.035 b	0.629 ± 0.025 b	0.257 ± 0.062 b	0.851 ± 0.039 b	0.560 ± 0.029 b	0.013 ± 0.007 b
Pea	0.562 ± 0.087 b	0.616 ± 0.040 b	0.290 ± 0.035 ab	0.824 ± 0.061 bc	0.544 ± 0.048 b	0.015 ± 0.007 b
Cabbage	0.649 ± 0.102 a	0.538 ± 0.040 c	0.328 ± 0.029 a	0.803 ± 0.020 c	0.452 ± 0.066 c	0.014 ± 0.005 b
Fennel	0.542 ± 0.038 b	0.621 ± 0.031 b	0.326 ± 0.065 a	0.806 ± 0.037 c	0.549 ± 0.036 b	0.029 ± 0.026 a
Olive	0.395 ± 0,066 c	0.719 ± 0.016 a	0.172 ± 0.026 bc	0.898 ± 0.019 b	0.666 ± 0.019 a	0.010 ± 0.003 bc
Control (no plants)	0.381 ± 0.040 c	0.726 ± 0.015 a	0.102 ± 0.016 d	0.938 ± 0.019 a	0.675 ± 0.018 a	0.005 ± 0.002 c

**Table 3:** Litter organic carbon (LOC), litter total nitrogen (LTN), and LOC/LTN ratio from the green tea and red tea bags; soil organic carbon (SOC), soil total nitrogen (STN), SOC/STN ratio, and soil pH. Each value represents the mean (±SD) of five replicates (composite soil samples; *n* = 5) for each species. The values followed by different letters (a–d) are statistically different (*p* ≤ 0.01) within columns.

	Green Tea			Red Tea			Soil			
Plant Species	LOC (g · kg^−1^)	LTN (g · kg^−1^)	LOC/LTN	LOC (g · kg^−1^)	LTN (g · kg^−1^)	LOC/LTN	SOC (g · kg^−1^)	STN (g · kg^−1^)	SOC/STN	pH
Before incubation/planting	44.64 ± 2.03 a	2.65 ± 0.43 b	16.85 ± 1.35 a	43.45 ± 3.54 a	3.23 ± 0.20 a	13.45 ± 1.92 bc	38.54 ± 0.98 a	3.20 ± 0.20 ab	12.04 ± 0.87 a	7.15 ± 0.11 a
Broad bean	28.09 ± 4.21 b	3.05 ± 0.04 a	9.21 ± 1.90 c	36.97 ± 1.21 b	3.56 ± 0.43 a	10.38 ± 0.23 c	31.96 ± 3.42 c	3.55 ± 0.20 a	9.00 ± 1.12 d	6.62 ± 0.09 b
Pea	27.49 ± 6.78 b	2.88 ± 0.14 a	9.54 ± 0.43 c	35.81 ± 0.73 b	3.25 ± 0.02 a	11.02 ± 2.34 c	31.44 ± 4.53 c	3.62 ± 0.53 a	8.69 ± 0.86 d	6.68 ± 0.09 b
Cabbage	24.03 ± 2.61 c	1.71 ± 0.04 c	14.05 ± 2.11 ab	34.90 ± 0244 b	2.04 ± 0.04 c	17.11 ± 2.86 a	28.45 ± 2.90 d	2.83 ± 0.38 b	10.05 ± 0.05 c	6.59 ± 0.12 b
Fennel	27.70 ± 3.00 b	1.80 ± 0.25 c	15.39 ± 0.83 a	35.01 ± 3.54 b	2.21 ± 0.09 c	15.84 ± 2.38 ab	31.62 ± 0.77 c	2.91 ± 0.19 c	10.87 ± 2.45 c	6.28 ± 0.04 c
Olive	32.09 ± 2.39 b	2.12 ± 0.28 b	15.14 ± 0.54 a	39.01 ± 3.63 b	2.67 ± 0.29 b	14.61 ± 1.55 b	35.41 ± 1.41 b	2.67 ± 0.36 c	13.26 ± 2.32 a	6.90 ± 0.14 ab
Control (no plants)	32.41 ± 2.09 b	2.50 ± 0.33 b	12.96 ± 1.03 b	40.76 ± 4.32 ab	2.65 ± 0.17 b	15.38 ± 2.45 ab	35.69 ± 1.51 b	3.09 ± 0.27 ab	11.55 ±1.64 bc	7.10 ± 0.05 a

## Supplementary Materials

The following are available online at https://www.mdpi.com/2223-7747/9/10/1338/s1, Table S1: Dry weight and main length of shoots and roots, and root density of different plant species measured after 90 days of cultivation. Each value represents the mean (±SD) of five replicates (plants; *n* = 5) for each species. The values followed by different letters (a–e) are statistically different (*p* ≤ 0.01) within columns. Table S2: Pearson correlation coefficients (*R*) among the measured parameters from the five plant species. *: significant difference at *P* < 0.05; **: significant difference at *P* < 0.01. DW = dry weight; *S* = stabilization factor; *k* = decomposition rate constant; SOC = soil organic carbon; STN = soil total nitrogen; TBC = total bacterial count; TFC = total fungal count. Figure S1: Root portions analyzed by SmartRoot software: (a) broad bean, (b) pea, (c) cabbage, and (d) fennel. Scale bar = 1 cm. Figure S2: Plants of (a) fennel, (b) broad bean, and (c) cabbage used during the experiment. Ruler length = 15 cm. Figure S3: The green (left) and red (right) tea bags recovered from soil after 90 days.
