# Supplementary material for: Structural and Functional Organization of the Root System: A Comparative Study on Five Plant Species
Source: Plants (Basel). 2020 Oct 10;9(10):1338. doi: 10.3390/plants9101338 (PMC7601878; doi:10.3390/plants9101338)
Supplement: Supplementary file 1 [file plants-09-01338-s001.pdf]

Supplementary Materials

Table S1. Dry weight and main length of shoots and roots, and root density of different plant species measured after 90 days of cultivation. Each value represents the mean (± SD) of five replicates (plants; $n = 5$) for each species. The values followed by different letters (a-e) are statistically different ($p \leq 0.01$) within columns.

Plant species	Shoot DW (g)	Root DW (g)	Root/shoot DW	Main shoot length (cm)	Main root length (cm)	Main root/shoot length
Broad bean	75.40 ± 4.45 b	23.66 ± 2.25 b	0.31 ± 0.07 b	56.90 ± 9.36 c	13.23 ± 4.06 c	0.23 ± 0.04 b
Pea	22.87 ± 3.33 e	7.25 ± 1.36 d	0.32 ± 0.04 b	78.54 ± 5.18 b	14.70 ± 2.66 c	0.19 ± 0.04 c
Cabbage	31.36 ± 3.28 d	11.26 ± 2.49 c	0.36 ± 0.06 b	39.40 ± 6.99 d	37.92 ± 5.13 b	0.96 ± 0.13 a
Fennel	45.01 ± 8.92 c	7.79 ± 1.57 d	0.17 ± 0.08 c	68.65 ± 7.29 b	12.90 ± 3.01 c	0.19 ± 0.06 c
Olive	1241.76 ± 121.73 a	829.21 ± 110.80 a	0.68 ± 0.10 a	162.71 ± 18.46 a	43.01 ± 5.31 a	0.26 ± 0.05 b

Table S2. Pearson correlation coefficients (*R*) among the measured parameters from the five plant species studied. *: significant difference at $P < 0.05$; **: significant difference at $P < 0.01$. DW = dry weight; *S* = stabilization factor; *k* = decomposition rate constant; SOC = soil organic carbon; STN = soil total nitrogen; TBC = total bacterial count; TFC = total fungal count.

	**Root DW**	**Total root length**	**Root tips**	**Average root diameter**	**Total root surface area**	***S***	***k***	**SOC**	**STN**	**Soil pH**	**TBC**	**TFC**
**Root DW**	1.00											
**Total root length**	–0.82*	1.00										
**Root tips**	–0.75**	0.72**	1.00									
**Average root diameter**	0.62	0.27	0.24	1.00								
**Total root surface area**	0.69**	0.70**	0.55	0.33	1.00							
S	0.88*	0.90*	0.58	0.41	0.76**	1.00						
k	0.72**	0.71**	0.62	0.16	0.82**	–0.74**	1.00					
**SOC**	0.65	0.80**	0.53	–0.15	0.69	–0.89*	0.68**	1.00				
**STN**	0.61	0.77**	0.60	0.18	0.55	–0.72**	0.66	0.88*	1.00			
**Soil pH**	0.71**	0.82*	0.64	0.47	0.94*	–0.57	0.73**	0.68**	0.65	1.00		
**TBC**	0.65	0.86*	0.39	0.60	0.92*	–0.78*	0.87*	0.74**	0.78**	0.51	1.00	
**TFC**	0.72**	0.59	0.53	0.70**	0.73**	–0.68**	0.88*	0.74**	0.90*	0.57	0.88*	1.00

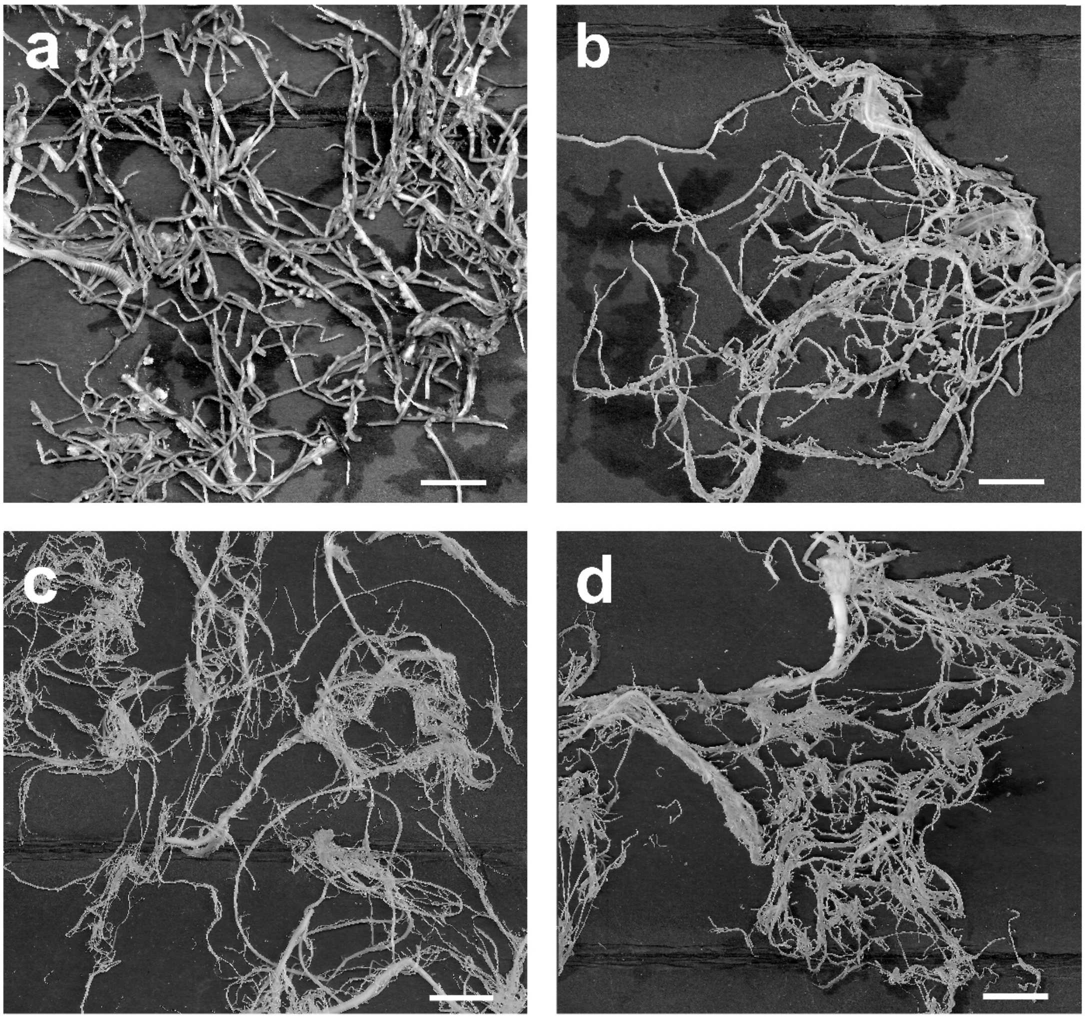

Figure S1. Root portions analysed by SmartRoot software: (a) broad bean, (b) pea, (c) cabbage, and (d) fennel. Scale bar = 1 cm.

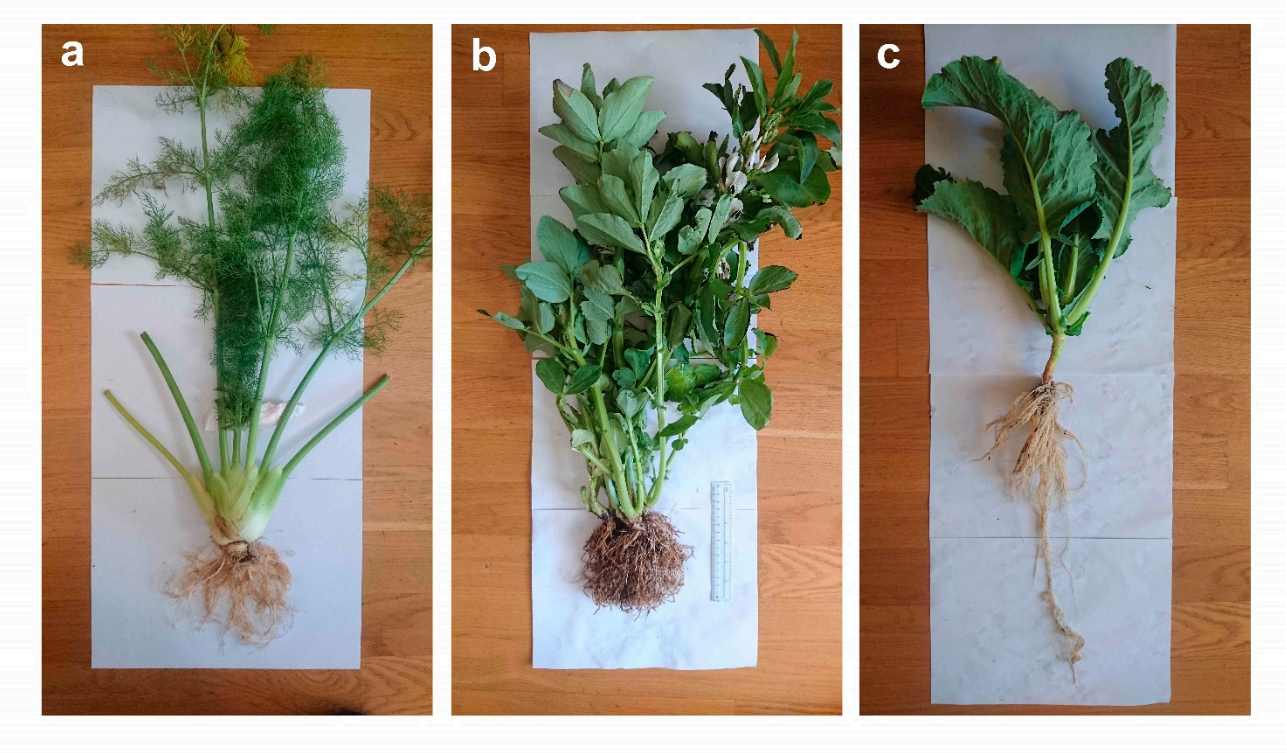

Figure S2. Plants of (a) fennel, (b) broad bean, and (c) cabbage used during the experiment. Ruler length = 15 cm.

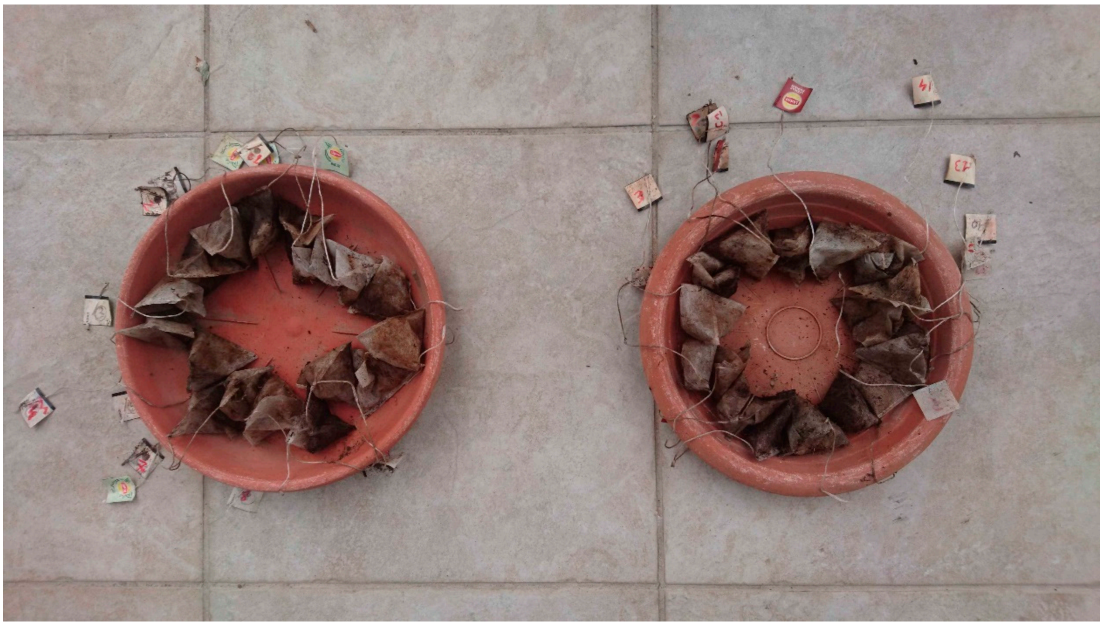

Figure S3. The green (left) and red (right) tea bags recovered from soil after 90 days.
